# Hydrogen production from methanol aqueous solution by ZnO/Zn(OH)_2_ macrostructure photocatalysts

**DOI:** 10.1039/c8ra00943k

**Published:** 2018-03-22

**Authors:** Yilun Wu, Shan Zeng, Yanhui Dong, Yunhao Fu, Hang Sun, Shengyan Yin, Xingyuan Guo, Weiping Qin

**Affiliations:** State Key Laboratory of Integrated Optoelectronics, College of Electronic Science and Engineering, Jilin University Changchun Jilin 130012 China syyin@jlu.edu.cn wpqin@jlu.edu.cn; Key Laboratory of Bionic Engineering (Ministry of Education), College of Biological and Agricultural Engineering, Jilin University Changchun Jilin 130022 P. R. China

## Abstract

Photocatalytic H_2_ generation was studied for a series of ZnO/Zn(OH)_2_ macrostructure photocatalysts. Different ZnO/Zn(OH)_2_ macrostructures were prepared through a one-step hydrothermal method by adjusting the pH values of the solution and the concentration of dodecyl sulfate. Three different morphologies of the ZnO/Zn(OH)_2_ macrostructure were synthesized and studied using SEM and XRD. The reflectance spectra revealed that the cone shaped ZnO/Zn(OH)_2_ macrostructure (ZnO-C) had the lowest reflectivity of UV light. It was found that the photoelectronic properties depend on the morphology of the ZnO/Zn(OH)_2_ macrostructures. The photocatalytic activity of these ZnO/Zn(OH)_2_ macrostructure hybrids (about 0.070 mmol g^−1^ h^−1^) were higher than that observed for ZnO nanorods (0.050 mmol g^−1^ h^−1^). These results suggest the substantial potential of metal oxide materials with macrostructures in photocatalytic water splitting applications.

## Abstract 2

ZnO/Zn(OH)_2_ macrostructure photocatalysts prepared using a one-step hydrothermal method demonstrated highly efficient photocatalytic hydrogen production.

## Graphical abstract

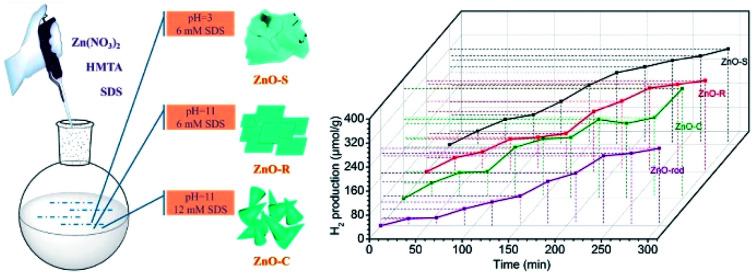

## 1. Introduction

Photo-derived water splitting for hydrogen generation is considered a promising method to generate an alternate and renewable energy source to relieve the energy crisis. It converts solar energy, the nearly infinite source of energy, into carbon-free chemical energy, which can be used and stored easily with clean processes, *i.e.*, hydrogen.^1–3^ Since the first photo-assisted electrochemical water splitting experiment was conducted in 1972, many new materials and approaches have been developed to improve the photocatalytic hydrogen generation efficiency,^4^ such as changing the reaction conditions, applying bias voltage and introducing a semiconductor. A semiconductor whose energy bands are matched with the photocatalytic reaction system acts as the photocatalyst and can significantly improve the feasibility of solar energy transfer. Moreover, cheap, efficient, and stable semiconductor photocatalysts have considerable potential to generate hydrogen on a large scale.^5^ Some semiconductors, particularly wide band gap materials, such as TiO_2_, ZnO, Fe_2_O_3_, WO_3_, ZnS, CdS and g-C_3_N_4_, have been proven to excite the electrons to reduce H^+^ and produce hydrogen from water when exposed to sunlight.^4,6–14^ These materials are cheap, abundant and environmentally friendly and can be prepared easily and cost-effectively using the hydrothermal growth method or electro-deposition method.^15,16^ However, there are also some disadvantages with these materials, which limit the conversion efficiencies of the solar-to-hydrogen transfer process, such as low conductivity, short diffusion length, poor charge conduction between the reagent and photocatalyst, low light energy utilization and poor surface evolution kinetics.^17,18^ In order to avoid these disadvantages, one possible way is to fabricate these materials with some special structures, which can increase the reaction surface area, reduce carrier recombination, improve the utilization of light energy and charge transfer.^19–21^ Therefore, many nano/micro structures of these semiconductors have been reported and they also exhibit good performance in the photocatalytic application field.^15,22,23^

In recent years, with a wide band gap, non-toxic nature, excellent photosensitivity property, and large exciton binding energy, ZnO is considered as a good photocatalyst, which may be a reliable candidate for solving energy problems.^22,24^ However, the photogenerated electrons and holes combine readily during the photocatalytic process due to the strong exciton binding. Thus, it is essential to develop a new type of ZnO photocatalyst, which can accomplish the effective separation of the photo-generated exciton pairs.^25^ For this aim, numerous approaches have been investigated, such as doping with other elements, constituting a heterojunction and introducing new micro/nano structures.^21,26,27^ As mentioned above, the introduction of micro/nano structures is a simple and promising method to promote photocatalytic hydrogen production due to their enhanced surface area, improved light absorption and increased charge transfer efficiency.^21,28,29^ Thus, the construction of certain structures of the ZnO material is a promising route for photocatalytic research.

Since the discovery of graphene, a large number of two-dimensional (2D) materials have been prepared, such as nitride, sulphide, selenide and telluride.^30–35^ These 2D materials exhibit large specific surface areas, excellent mechanical properties and unique electrical characteristics. Moreover, how to produce high quality layered materials on a large scale is a considered step for the development of 2D materials. A cost-efficient procedure to prepare mesoporous ZnO is the high temperature decomposition of layered Zn(OH)_2_.^36^ Nevertheless, after the calcination process, the layered structure was destroyed and the photocatalytic properties were similar to the ZnO nanoparticles.^36^ Thus, how to fabricate 2D ZnO materials and make further use of the advantages of such materials is worth studying.

In this article, we investigated the preparation and photocatalytic properties of layered and hollow cone ZnO/Zn(OH)_2_ macrostructure hybrids. This macrostructure configuration as well as the chemical procedure is expected to favour the formation of interfaces among the different components. Three ZnO/Zn(OH)_2_ macrostructures were prepared by adjusting the pH values and the amount of the surfactant. In these macrostructures, layered Zn(OH)_2_ acts as a structural component, which enhances the surface area and reinforces the structural strength, while ZnO acts as the functional component, which can utilize light energy and generate hydrogen. To perform a clear comparison of these photocatalysts, all of these products were assayed for photocatalytic H_2_ production using methanol as a model sacrificial reagent. These results suggest the substantial potential of the ZnO material with the aforementioned macrostructures in photocatalytic hydrogen evolution applications.

## 2. Experimental section

### 2.1 Materials

Methanol (CH_3_OH, 99.5%), ethanol (C_2_H_5_OH, 99.7%), hydrochloric acid (HCl, 36–38%), sodium hydroxide (NaOH, 96.0%), and sodium dodecyl sulfate (SDS, 99.9%) were obtained from Beijing Chemical Works. Zinc nitrate (Zn(NO_3_)_2_, 99.0%) and hexamethylenetetramine (HMTA, 99.0%) were purchased from Tianjin Guangfu Fine Chemical Research Institute and Tianjin Huadong Reagent Works, respectively. Deionised water (resistance ≥ 18.25 MΩ cm^−1^, PINGCHENG pure technology Co. LTD) was used in all the experiments. Nafion solution (5 wt%) was obtained from DuPont company.

### 2.2 Preparation of the ZnO/Zn(OH)_2_ macrostructures

The ZnO/Zn(OH)_2_ macrostructures were prepared thorugh a hydrothermal growth method. Briefly, 0.4 mmol Zn(NO_3_)_2_, 0.4 mmol HMTA and an appropriate amount of SDS were added to a 250 mL flask and dissolved in a suitable amount of water. The total volume of the solution was controlled to 100 mL and the solution pH was adjusted to a certain value (dependent on the macrostructures of the products) by the addition of NaOH or HCl. The above mixture was kept at 90 °C for 1.5 h with mild stirring (200 rpm) and then, the reaction was stopped. The resultant solution gradually became turbid and the aqueous solution showed some glossy stripes on swaying the flask, which indicated that the growth of the ZnO/Zn(OH)_2_ macrostructures was completed.

The preliminary solution was centrifuged and the aqueous supernatant was removed. The resultant ZnO/Zn(OH)_2_ macrostructure precipitate was successively washed several times with water and ethanol to make sure that the surfactant was adequately removed. Finally, the ZnO/Zn(OH)_2_ macrostructure precipitate was dried at 60 °C overnight and the pure ZnO/Zn(OH)_2_ sample was obtained.

These ZnO/Zn(OH)_2_ macrostructures with three different morphologies, namely, sheet-shaped (ZnO-S), square-shaped (ZnO-R) and cone-shaped (ZnO-C) were synthesized using the above procedure. All of the ZnO/Zn(OH)_2_ macrostructures were prepared with the same starting reagents, while the morphologies were controlled by the auxiliary agent. The primary reaction mixture was prepared with formula listed in Table 1.

**Table 1:** The initial reaction ratio of the reactants used for the different ZnO/Zn(OH)_2_ macrostructure samples

Materials	Samples		
	ZnO-S	ZnO-R	ZnO-C
Zn(NO_3_)_2_	4 mM	4 mM	4 mM
HMTA	4 mM	4 mM	4 mM
SDS	6 mM	6 mM	12 mM
pH	3	11	11

In addition, ZnO nanorods (ZnO-rod) were synthesized without SDS for the control experiment. The initial reaction ratio of Zn(NO_3_)_2_ and HMTA was same as that for the other samples.

### 2.3 Photocurrent measurements

A typical three-electrode system was used for the photocurrent measurements performed on an electrochemical station (CHI 660E). The ZnO/Zn(OH)_2_ samples were used as the working electrode, Pt electrode was used as the counter electrode and Ag/AgCl electrode (3.0 M KCl) was used as the reference electrode. Additionally, a Na_2_SO_4_ (0.5 M) solution was used as the electrolyte and a high pressure Xe lamp (300 W, CEAULIGHT, Co., Ltd.) was used as the solar simulator source. The light intensity was 20 mW cm^−2^ in all the experiments. The area of the light spot was about 30 cm^2^. The working electrode was prepared using a literature method.^37^ Typically, 10 mg of the sample was dispersed in water and then coated on ITO glass with the area about 1 cm^2^. After being dried at room temperature, a thin layer of Nafion was coated on the surface of the sample and the resultant material used as the working electrode.

Electrochemical impedance spectroscopy (EIS) measurements were conducted in the abovementioned three-electrode system using a CHI 660E electrochemical workstation with a Pt counter electrode and Ag/AgCl reference electrode. A quartz cell was introduced to allow the incident light from the source (solar simulator 300 W Xe lamp, CEAULIGHT, Co., Ltd.) to irradiate the working electrode. A 0.5 M Na_2_SO_4_ aqueous solution was used as the electrolyte. AC perturbations of 5 mV amplitude were applied and the frequency ranged from 0.01 Hz to 100 kHz.

### 2.4 Photocatalytic activity

The photocatalytic H_2_-production was evaluated using a photocatalytic activity evaluation system (CEL-SPH2N), which was purchased from CEAULIGHT. A high pressure Xe lamp (300 W, CEAULIGHT, Co., Ltd.) was used as the light source. The light intensity was set at 20 mW cm^−2^ and the light spot was about 30 cm^2^.

In this photocatalytic H_2_ production test, 50 mg of the photocatalyst was suspended in 50 mL of an aqueous solution, containing 20% methanol. The reaction system was continuously cooled by externally circulating refrigerated water to keep the temperature of the reaction system unchanged throughout the test. Prior to irradiation, the reaction system should be maintained in the vacuum environment. For adequately dispersing the photocatalyst, continuous magnetic stirring was applied during the entire experiment. The hydrogen was analysed using a gas chromatograph (GC-7920, CEAULIGHT, 5 Å molecular sieve column, TCD detector, N_2_ carrier gas).

### 2.5 Characterization methods

Scanning electron microscopy (SEM) images were obtained on an HITACHI TM-1000 apparatus. The crystalline structures of the three different ZnO/Zn(OH)_2_ macrostructure samples, namely, ZnO-S, ZnO-R, and ZnO-C were measured on a SHIMADZU XRD-6100 diffractometer with Cu Kα radiation at a wavelength of 1.5406 Å and the data were collected from 10° to 70°. The reflectance spectra covering a broad wavelength range of 200–800 nm were obtained on a SHIMADZU UV-3600 apparatus.

## 3. Results and discussion

**Fig. 1:**
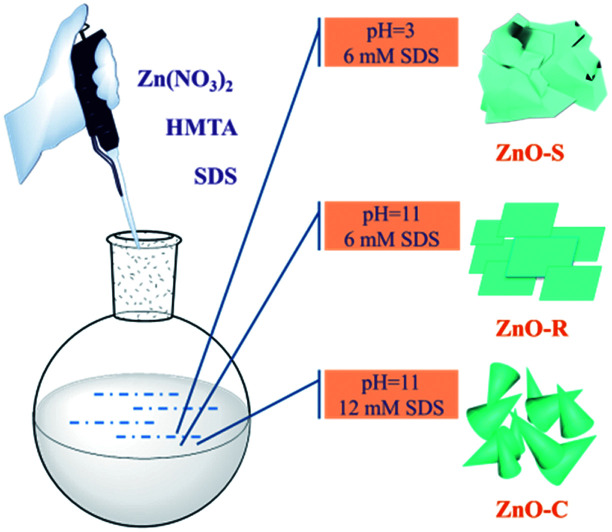
Preparation of the ZnO/Zn(OH)_2_ macrostructures.

**Fig. 2:**
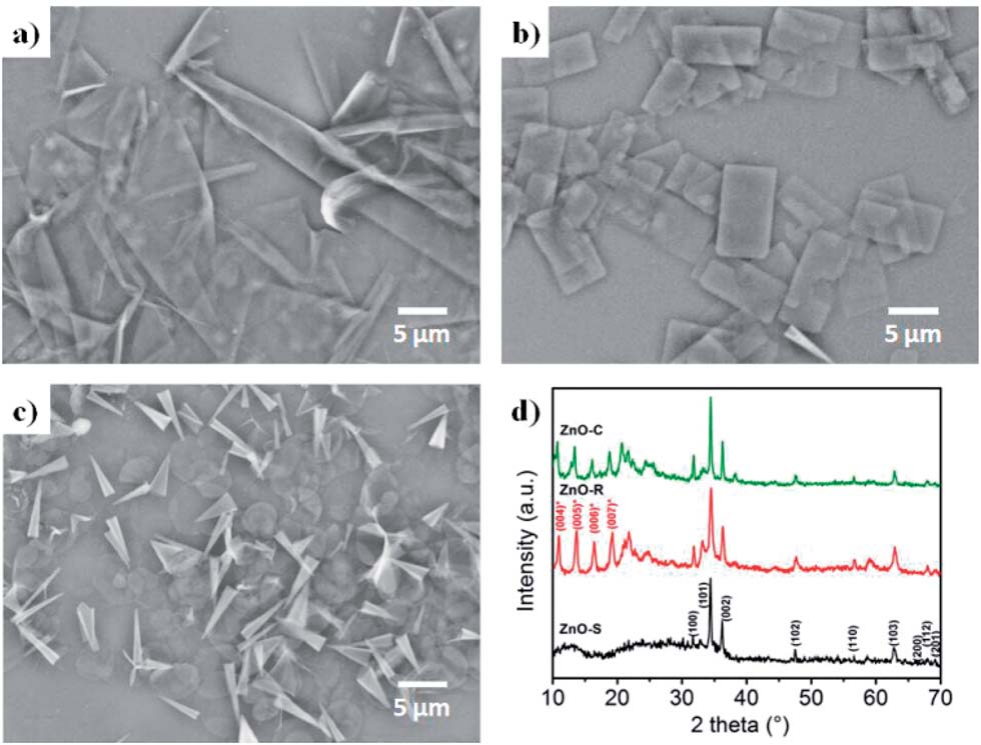
Configuration of the ZnO/Zn(OH)_2_ macrostructures. SEM images of (a) ZnO-S, (b) ZnO-R and (c) ZnO-C. (d) XRD patterns of the ZnO/Zn(OH)_2_ macrostructures.

The ZnO/Zn(OH)_2_ macrostructures were synthesized *via* a hydrothermal method and the synthesis process is shown in Fig. 1. The HMTA and Zn(NO_3_)_2_ were used at a concentration of 4 mM. Moreover, the concentration of SDS surfactant and pH of the solution were differentiated to control the morphology of products. Specifically, the precursor solution of ZnO-S sample contained 6 mM SDS and its pH value was 3, while the ZnO-R and ZnO-C samples were prepared in an alkaline solution (pH = 11) with 6 mM and 12 mM SDS, respectively. The corresponding morphologies of these ZnO/Zn(OH)_2_ macrostructures were studied by SEM and XRD. As shown in Fig. 2a, ZnO/Zn(OH)_2_ exhibits some large and thin sheets, which were prepared with an SDS concentration of 6 mM and pH value of 3. Lower pH values were also applied. Actually, the mild acidity of the reaction solution is evidently beneficial to the formation of the sheet macrostructure. Moreover, hydrogen ions severely inhibit the production of ZnO/Zn(OH)_2_ with no product being obtained at pH < 2. In order to balance the production yield and quality, the appropriate pH value was found to be 3. The diameter of the ZnO/Zn(OH)_2_ macrosheets (ZnO-S) was nearly 20 μm (Fig. 2a). The synthesis of the ZnO/Zn(OH)_2_ macrostructures can be promoted under alkaline conditions (pH = 11). When the concentration of SDS was 6 mM, the ZnO/Zn(OH)_2_ was formed with regular square-shaped morphology (ZnO-R) with the size of about 10 μm × 4 μm (Fig. 2b). When the SDS concentration was increased to 12 mM, the morphology of ZnO/Zn(OH)_2_ changed to spherical and conical (ZnO-C, as shown in Fig. 2c).

The crystalline structures of the ZnO/Zn(OH)_2_ products were obtained and revealed the crystal formation of the composite. As shown in Fig. 2d, the diffraction peaks of ZnO-S at 31.8°, 34.4°, 36.2°, 47.5°, 56.6°, 62.9°, 67.9°, 68.1° and 69.1° were indexed to the (100), (002), (101), (102), (110), (103), (200), (112) and (201) planes of wurtzite ZnO (JCPDS 36-1451), respectively. No more peaks of the sphalerite phase can be found in this pattern. There was a broad diffraction at 12°, which may be the peak of Zn(OH)_2_ with poor crystallinity. The ZnO-R and ZnO-C prepared in the alkaline solution showed diffraction peaks at 10.9°, 13.7°, 16.4° and 19.2°, which were indexed to the (004), (005), (006), and (007) planes of Zn(OH)_2_, respectively.^36^ The diffraction peaks of ZnO were still observed, which indicated that the ZnO-R and ZnO-C were composites of ZnO and Zn(OH)_2_. Generally, the ratio of ZnO and Zn(OH)_2_ in the three samples could be roughly revealed in their XRD patterns. The composite prepared under acidic conditions possess more of the ZnO component and those prepared under alkaline conditions possess more of the Zn(OH)_2_ component, which affects the properties of these materials.

In such a precursor solution, HMTA acts as an alkaline releaser and promotes the hydrolysis of Zn^2+^ into ZnO. During the growth of the ZnO crystals, large amounts of Zn^2+^ were exposed in the (002) facet and then absorbed large amounts of dodecyl sulfate (DS^−^) ions, which obstruct any further crystallization in the [002] direction. The ZnO crystals tend to expand to large and thin sheets such as ZnO-S and ZnO-R. Furthermore, upon increasing the amount of DS^−^, the crystal growth in the other directions was remarkably blocked. Thus, in this situation, the product we obtained was regular and thicker and could even be rolled into some hollow cones (ZnO-C). Moreover, the specific pH environment of the precursor solution enhanced the morphology control process. Actually, the morphology of the product would be unpredictable in a neutral precursor solution. During the ZnO growth process, a part of the ZnO^2+^ can combine with OH^−^ in the solution and produce Zn(OH)_2_, which is evidently affected by the OH^−^ concentration. Consequently, the acidic conditions provide a low OH^−^ density environment and the ZnO could grow larger as well as possess a small amount of Zn(OH)_2_ (ZnO-S). Correspondingly, the alkaline conditions are enriched with OH^−^ and facilitate ZnO growth. Moreover, a large amount of Zn(OH)_2_ is introduced in the product, which causes the cleavage of the crystals and the formation of some rectangular pieces (ZnO-R).

**Fig. 3:**
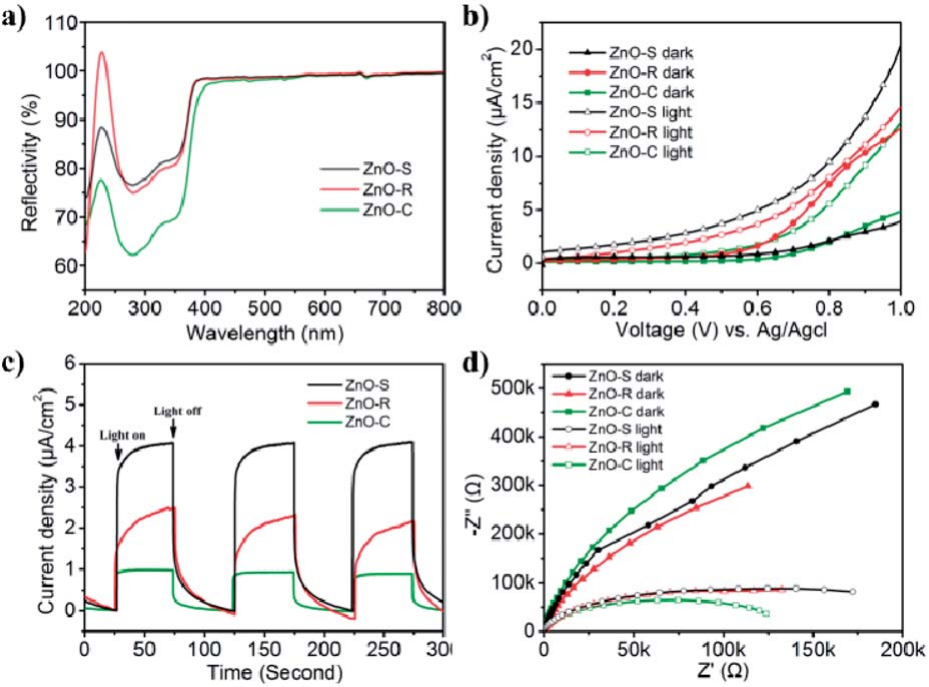
Diffuse reflectance spectra and photoelectrochemical properties of ZnO-S (black), ZnO-R (red), and ZnO-C (green): (a) reflectance spectra; (b) *I*–*V* characteristics of the ZnO/Zn(OH)_2_ macrostructures with and without light irradiation; (c) current *versus* time under chopped irradiation at a bias voltage of 0.7 V (*vs.* Ag/AgCl); (d) EIS responses in the absence and presence of light irradiation.

The light utilization efficiency is an important part in the photocatalytic process. In order to study the absorbance of the ZnO/Zn(OH)_2_ composites, the standard absolute hemispherical reflectance spectra were recorded using an integrating sphere at normal incidence. The band gap of pure ZnO is about 3.2 eV and the corresponding wavelength is around 380 nm.^26^ As shown in Fig. 3a, these samples reflect almost the entire visible light region (*λ* > 380 nm) and showed certain reflection properties in the UV region (250 nm < *λ* < 380 nm). This result is consistent with the band gap of pure ZnO, *i.e.*, only when the photon energy is higher than 3.2 eV, the incident light can be absorbed. The ZnO-S and ZnO-R reflect about 75% of UV light owing to their relatively flat structure. ZnO-C reflected 62% of the incident light and the reduced reflection effect can be attributed to its cone structure. Thus, we can infer that the structure of the cone shaped ZnO/Zn(OH)_2_ composite can enhance the light absorption.

The photoelectric activity of the ZnO/Zn(OH)_2_ macrostructures was studied using electrochemical tests. The photocurrent measurements were performed in an electrochemical system using a quartz cell and Na_2_SO_4_ (0.5 M) electrolyte. The ZnO/Zn(OH)_2_ composite deposited on the ITO glass acted as the working electrode, Pt wire as the counter electrode and an Ag/AgCl electrode as the reference electrode. As shown in Fig. 3b, the *I*–*V* curves obtained in the dark and upon light irradiation were compared for ZnO-S, ZnO-R and ZnO-C. All the samples showed almost no current without light irradiation at low bias voltage, which indicated that no leakage current was observed in the ZnO/Zn(OH)_2_ macrostructures under dark conditions. At high bias voltage, the ZnO-S and ZnO-C samples also showed a current density of 4 μA cm^−2^ and 5 μA cm^−2^, respectively, while ZnO-R showed a current density of 13 μA cm^−2^ under dark conditions. An increase in current was observed when these samples were irradiated by the incident light. Upon light irradiation, ZnO-S produced a photocurrent starting at 0 V (*vs.* Ag/AgCl reference electrode) that continued to increase to 20 μA cm^−2^ at 1.0 V (Fig. 3b). More interestingly, saturation of photocurrent was not observed even at high bias voltage, which indicated the efficient charge separation of the inner hybrid materials. Actually, the promotion of the photocurrent was caused by the number of newborn holes and electrons, which were excited by illumination. The ZnO-R and ZnO-C also displayed good photocurrent generation. The photocurrent density of the ZnO-R and ZnO-C samples increased to 15 μA cm^−2^ and 13 μA cm^−2^ at 1.0 V, respectively. Their photocurrent was not saturated at 1.0 V, which was identical to ZnO-S. The photoelectric responses of ZnO-S, ZnO-R and ZnO-C were observed using chronoamperometry. The potential of the working electrode during the test was set at 0.7 V (*vs.* Ag/AgCl). As shown in Fig. 3c and a fast and stable photocurrent of 4 μA cm^−2^ was observed for ZnO-S with the incident light switched-on and switched-off. ZnO-R showed 2 μA cm^−2^ and ZnO-C showed 1 μA cm^−2^ under the same experiment conditions. All of these records were significantly smaller than the current density of ZnO-S. The photoresponse of these samples in the ON/OFF cycle was completely reversible and the response of ZnO-C was sharper than that of the other samples. The significant photocurrent generation of ZnO-S, ZnO-R and ZnO-C indicated that these samples showed a good separation of the generated electron–hole pairs, which was beneficial for photocatalysis. Considering the XRD results, ZnO-S possesses more of the ZnO component, which exhibits higher conductivity than Zn(OH)_2_.^38^ Moreover, the thin and large configuration of ZnO-S easily forms a tight connection among the single sheets and then creates numerous channels to transfer the carriers.^21^ Thus, ZnO-S can display the highest photocurrent. For the same reason, ZnO-R contains the charge channels, but its material conductivity is lower and hence, it presents moderate current density. ZnO-C hardly has the above advantages and hence presents the lowest current density.

Electrochemical impedance spectroscopy (EIS) was performed in the same system and can be used to study the charge transfer observed in the ZnO/Zn(OH)_2_ macrostructures. All of the photo-response data under dark and light conditions were plotted with Nyquist diagrams as shown in Fig. 3d. When comparing these curves, these samples exhibited differences in the arc radius. Normally, the arc radius of the EIS curve is related to the electron–hole separation and interfacial charge transfer processes, *i.e.*, a smaller arc radius implies better carrier transfer and effective charge separation.^21^ The arc radius of all the three samples was so large that it could only display a part of the circle. As shown in Fig. 3d, all the samples exhibited a much smaller arc radius under light irradiation than that under dark, which revealed the occurrence of the charge separation and transfer process. The ZnO-C EIS curve exhibited the largest and the smallest arc radius under the dark and light irradiation, respectively, indicating its high sensitivity to light, which is beneficial for its application as a light sensor.^39^ The EIS curve radius of ZnO-S was greater under light irradiation and smaller in the dark than that of the ZnO-C sample, which indicates that the ZnO-S had a weak response to light. ZnO-R displayed the dullest photo-response property, while its curve radius under light irradiation was nearly the same as that of ZnO-S. From the results of the photoelectrochemical tests, it can be inferred that these three ZnO/Zn(OH)_2_ macrostructures had noticeable photoelectric response properties, which might be advantageous in the photocatalysis field.

**Fig. 4:**
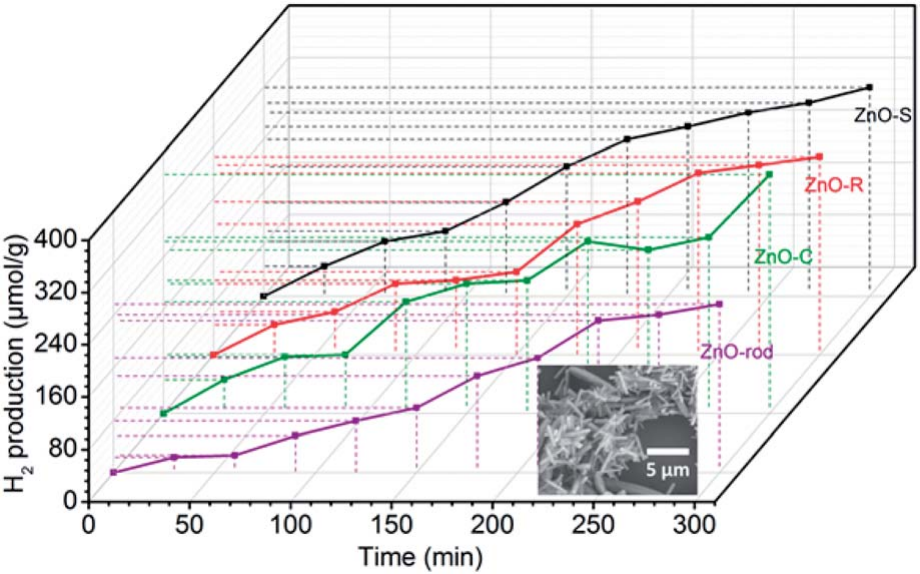
Photocatalytic H_2_-evolution activity of ZnO-S (black), ZnO-R (red), ZnO-C (green) and ZnO-rod (purple) tested in a methanol–water-mixed solution under simulated solar light irradiation (the inset is the SEM image of the ZnO-rod).

The photocatalytic H_2_ evolution of the ZnO/Zn(OH)_2_ macrostructures were evaluated in a mixed solution containing 20% methanol under simulated solar light irradiation (*λ* > 200 nm). As shown in Fig. 4, the H_2_ evolution rates of ZnO-S, ZnO-R and ZnO-C were 0.070 mmol g^−1^ h^−1^, 0.064 mmol g^−1^ h^−1^ and 0.069 mmol g^−1^ h^−1^, respectively. The ZnO-C and ZnO-S exhibited similar photocatalytic activity and slightly higher than that of ZnO-R. In addition, the H_2_ evolution of the pure ZnO nanorods under the same conditions was also studied. The H_2_-generation rate of the ZnO nanorods was 0.050 mmol g^−1^ h^−1^, which was lower than that of the ZnO/Zn(OH)_2_ macrostructures. These results suggest that the ZnO/Zn(OH)_2_ macrostructures can promote the generation and transfer of the photoelectrons, which is closely related to the H_2_ evolution process. Additionally, the hydrogen generation activities of some documented ZnO/Zn(OH)_2_ composite photocatalysts are shown in the Table 2. Although the rate of hydrogen generation in our system is not the best, the components in our system are the simplest, indicating the further application of the ZnO/Zn(OH)_2_ macrostructures.

**Table 2:** Hydrogen generation activities of some documented ZnO composite photocatalysts

Photocatalyst	Sacrificial reagents	Morphology	Light source	Wavelength	H_2_ generation rate^a^ [mmol h^−1^ g^−1^]	Reference
1.0% Pt/10% Zn(OH)_2_/Cd_0.3_Zn_0.7_S	Na_2_S/Na_2_SO_3_	Amorphous clumps (hundreds of nanometers)	LED (24.2 mW cm^−2^)	450 nm	0.436	40
(CdS–ZnS)/Ru (20%)	Methanol	Nanoparticle (40–340 nm)	Xe lamp (300 W)	>420^b^ nm	4.8 ± 0.3	41
Cd_0.75_Zn_0.25_S/ZnO/2D β-Zn(OH)_2_	Ethanol	Plate pieces (a few microns)	LED (32.7 mW cm^−2^)	450 nm	3.4	42
TiO_2_/CdS	Ethanol	Nanosphere (around 100 nm)	Xe lamp (300 W)	>420^b^ nm	0.033	43
Cd_*X*_Zn_1−*X*_S/Zn(OH)_2_	Ethanol	Plate pieces (a few microns)	LED (32.7 mW cm^−2^)	450 nm	2.256 (max)	38
ZnO/Zn(OH)_2_	Methanol	Irregular sheet (≈20 μm), rectangle & cone (≈5 μm)	Xe lamp (300 W)	>200 nm	0.07	This work

a The values are calculated according to the data reported in the literature.

b Placed in front of a 300 W Xe lamp with a 420 nm cut-off filter.

The ZnO/Zn(OH)_2_ macrostructures possess higher photocatalytic activity than that of the pure ZnO nanorods due to their special morphologies and crystal structures. Actually, the ZnO with the flake-shape promotes the carrier mobility of ZnO because the photo-generated electrons and holes can move to the crystal surface and react with the solution before they are recombined. Furthermore, the most exposed facet in these ZnO crystals is the (002) facet, which gathered a large amount of cations and exhibits strong polarity. It has been reported that the (002) facet possess higher activity than that of the (100) facet.^36^ Thus, we improved the photocatalytic power of ZnO by changing its crystal surface composition. Actually, due to the difference in their main shape and ZnO/Zn(OH)_2_ ratio, these ZnO/Zn(OH)_2_ composites with three different morphologies also exhibit photocatalytic nuances. The ZnO-S possess more ZnO ratio while the ZnO-C absorbs more incident light. These advantages make the H_2_ production ratio of ZnO-S and ZnO-C is higher than that of ZnO-R.

**Fig. 5:**
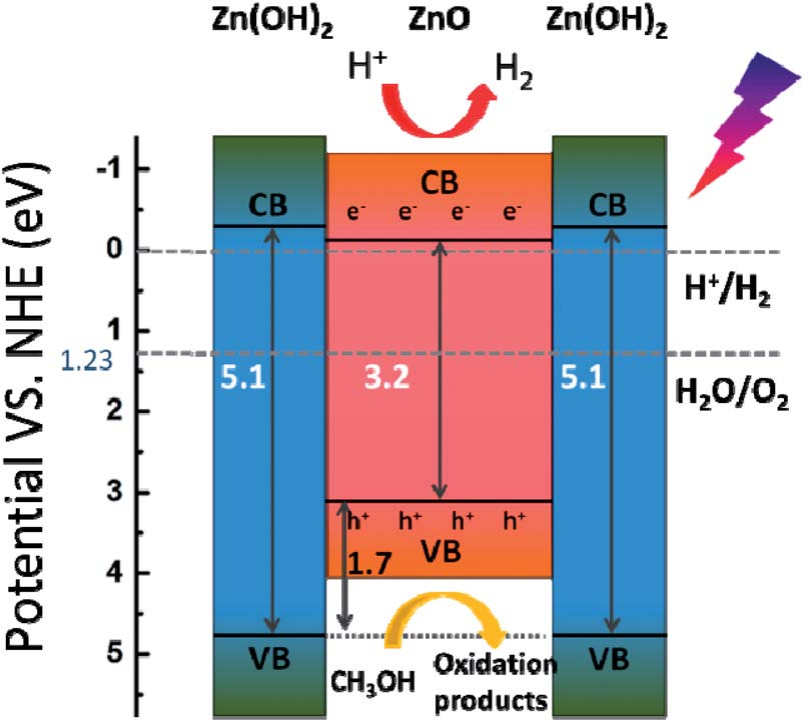
Schematic illustration of the electron–hole pair separation and transfer in the ZnO/Zn(OH)_2_ macrostructure and the proposed mechanism of photocatalytic H_2_ evolution under simulated solar light. CB = conduction band, VB = valence band, and h^+^ = hole.

Based on these results, the photocatalytic H_2_-evolution process of the ZnO/Zn(OH)_2_ macrostructures could be caused by the electron–hole pair separation and transfer. In the ZnO/Zn(OH)_2_ macrostructures, the ZnO nanocrystals are trapped in the Zn(OH)_2_ framework and form a heterojunction. A typical energy level graph of such contact is shown in Fig. 5. The corresponding light energy in the entire photocatalytic experiment is about 6.2 eV (*λ* > 200 nm), which is larger than the band gap of ZnO (3.2 eV) and Zn(OH)_2_ (5.1 eV). However, only a minor part (<1%) of the incident light from the source possesses sufficient energy to excite the electrons in the VB of Zn(OH)_2_. Moreover, the electrons in the VB of ZnO, particularly near the valance-band maximum, can be excited easily under the light irradiation. When the incident light wavelength is less than 380 nm, electrons obtain adequate energy to transfer into the conduction band (CB) and leave some holes in the VB,^27^ so that ZnO could play an important role in light absorption and generate the carriers. Moreover, the higher CB and lower VB of Zn(OH)_2_ promote the electrons and holes to flow into the ZnO. Thus, almost all of the photocatalytic process occurs in ZnO and the Zn(OH)_2_ maintains the structure of the composite. Charge carriers gathering in the ZnO can quickly move to the surface and increase the light current of the working electrode as shown in Fig. 3. Furthermore, the CB position of ZnO is slightly higher than the Normal Hydrogen Electrode (NHE), which forms a potential barrier between the electrons and H^+^ in the reaction solution. This potential barrier can promote the electron transition when H^+^ is attracted towards the interface, which is rich in electrons due to the electrostatic attractions. In the reaction system, H^+^ combines with the electrons from the ZnO/Zn(OH)_2_ macrostructure, due to which H_2_ is generated. Moreover, the holes in the VB act as strong oxidants to react with methanol because of their higher reactivity. The depletion of holes can protect the electrons in the CB from recombination to further improve H_2_ evolution.^26^

## 4. Conclusions

In this study, the ZnO/Zn(OH)_2_ macrostructures with three different morphologies, namely, ZnO-S, ZnO-R and ZnO-C were synthesised *via* a one-step hydrothermal method by adjusting the pH values and the amount of the surfactant. All of the samples exhibited uniform structures and noticeable photoelectric properties. These samples can absorb UV light and the ZnO-C sample exhibited higher anti-reflection than the other samples due to its uneven stacking configuration. The three ZnO/Zn(OH)_2_ macrostructures possess similar properties during photocatalytic H_2_ generation (about 0.070 mmol g^−1^ h^−1^) and exceed that observed for ZnO nanorods (0.050 mmol g^−1^ h^−1^). Considering these results, it is derivable that in the composites, Zn(OH)_2_ has a structural role of effectively increasing the light absorption and maintaining the thin configuration, while ZnO has a functional role of generating electrons and holes or producing hydrogen using light energy. It is conceivable that these ZnO/Zn(OH)_2_ macrostructures present a new approach for obtaining novel photocatalysts, which present advanced photocatalytic activity and effective H_2_ evolution.

## Conflicts of interest

There are no conflicts to declare.

## Supplementary Material
